# Attitudes to COVID-19 during the lockdown among university students in Russia and Uzbekistan

**DOI:** 10.1192/j.eurpsy.2022.1334

**Published:** 2022-09-01

**Authors:** E. Nikolaev, A. Aleksandrov, I. Poverinov, L. Niyazov

**Affiliations:** 1 Ulianov Chuvash State University, Social And Clinical Psychology Department, Cheboksary, Russian Federation; 2 Ulianov Chuvash State University, Department Of Public Law, Cheboksary, Russian Federation; 3 Ulianov Chuvash State University, Philosophy, Sociology And Pedagogy Department, Cheboksary, Russian Federation; 4 Bukhara State Medical Institute, Medical Chemistry Department, Bukhara, Uzbekistan

**Keywords:** Attitudes to COVID-19, Uzbekistan, university students, Russia

## Abstract

**Introduction:**

During the COVID-19 caused lockdown, when students had to study on-line, they became highly vulnerable to stress. How different were the attitudes of university students towards COVID-19 in such situation in different countries?

**Objectives:**

The goal is to determine the differences in attitudes to COVID-19 during the lockdown among university students in Russia and Uzbekistan

**Methods:**

In May 2020, there was an on-line survey of 163 students of both genders in Ulianov Chuvash State University in Russia and of 49 university students from Bukhara, Samarkand and Andijan in Uzbekistan. The instrument used was ‘Attitude towards COVID-19 Questionnaire’ (Nikolaev, 2020).

**Results:**

The comparison revealed that Uzbek students are more interested in the latest COVID-19 news than Russian ones (p=.0004), they also consider this pandemic as severe and dangerous for people (p=.0006), and think that governmental measures to fight coronavirus are adequate (p=.0008). Russian students in their turn, as compared with their Uzbek peers, feel highly concerned about the risk of their own infection (p=.00001), as well as the threat to their own life (p=.00546) and the life of their relatives and closest ones (p=.0005) as a result of coronavirus spread. In addition, Uzbek students regard themselves healthier than Russian ones (p=.0329). Students’ self-estimation of happiness does not differ (p=.0776).

**Conclusions:**

Differences in attitudes to COVID-19 among students are associated with more apparent socially oriented behavior of students from Uzbekistan, and more personality-oriented behavior of Russian students.

**Disclosure:**

No significant relationships.

